# Fatty acids promote uncoupled respiration via the ATP/ADP carrier in white adipocytes

**DOI:** 10.21203/rs.3.rs-5094089/v1

**Published:** 2024-09-25

**Authors:** Shannon Reilly, Ayla Aksu, Zane Zerbel, Preetveer Dhillon, Yosip Kelemen, Oluwafemi Gbayisomore, Serena Chen, Maryam Ahmadian

**Affiliations:** Weill Medical College of Cornell University; Weill Medical College of Cornell University; Weill Medical College of Cornell University; Weill Medical College of Cornell University; Weill Medical College of Cornell University; Weill Medical College of Cornell University; Weill Medical College of Cornell University; University of California SD

## Abstract

Adipocytes store energy as triglycerides, while mobilizing energy when needed via lipolysis. Triglyceride lipolysis releases fatty acids and glycerol into the circulation to fuel other tissues. However, a significant fraction of fatty acids released by lipolysis are retained within the white adipose tissue and handled by adipocytes. While some of these retained fatty acids are re-esterified in white adipocytes^1–6^, the a substantial amount undergo oxidative metabolism via a pathway regulated by the nongenomic effects of STAT3^7–10^. Here we report that fatty acids promote uncoupled oxidative metabolism in white adipocytes via the ATP/ADP carrier, contributing to thermogenesis and cold tolerance in obese thermoneutral-adapted mice, independent of brown adipose tissue and muscle activity. Our results suggest that uncoupled respiration in white adipocytes significantly contributes to whole-body energy expenditure and could be a promising target for obesity treatment.

## Introduction

Adipocytes are essential for the maintenance of metabolic health^11,12^. Excess nutrient storage as triglycerides (TGs) in adipocytes protects against ectopic lipid deposition and lipotoxicity. These lipids are hydrolysed during catabolic states such as starvation or cold exposure. Mobilization of TGs in the adipose tissue is prompted by catecholamine release from sympathetic nerve terminals, which activates lipolysis via b-adrenergic receptors. Fatty acid side chains are sequentially cleaved from the glycerol backbone during lipolysis, resulting in high intracellular fatty acid levels in white adipocytes^13^. While most of these free fatty acids are released from adipocytes providing fuel for other tissues, a fraction remains in the cell. How adipocytes handle this onslaught of fatty acids during active lipolysis is a fundamental question of metabolic importance.

Intracellular fatty acids generated by TG lipolysis can undergo b-oxidation or re-esterification^1,2^. In either case they must first be activated into fatty acyl-CoA. As professional lipid storing cells, white adipocytes typically esterify the vast majority of fatty acids taken up from the circulation in the fed state. During fasting, lipolysis results in increased intracellular fatty acids, which in turn promotes serine 727 phosphorylation of STAT3, and its interaction with and suppression of GPAT3, the first and rate limiting step in the glycerol lipid synthesis pathway utilized for TG synthesis^10^. This lipolysis-dependent inhibition of esterification diverts fatty acyl-CoAs into the oxidative pathway. Thus, elevated intracellular fatty acid levels during lipolysis drive oxidative metabolism in white adipocytes^9,10^.

Lipolytic activation of oxidative metabolism is associated with a decrease in mitochondrial membrane potential^7–9^. In brown adipocytes, the dissipation of the proton motive force is induced by fatty acid activation of Uncoupling protein 1 (UCP1) mediated proton leak^14^. Here we demonstrate that the ATP/ADP carrier (AAC also known as adenine nucleotide translocase ANT, encoded by *Slc25a4* and *Slc25a5*) is responsible for this uncoupling during lipolysis in white adipocytes. Like UCP1, AAC uncoupling activity is activated by fatty acids^15,16^. Furthermore, we demonstrate that lipolysis-driven oxidative metabolism and uncoupling contribute to thermogenesis and energy expenditure in obese thermoneutral-adapted mice, a physiologically relevant phenomenon which impacts whole-body energy balance.

## Results

### Lipolysis drives oxidative metabolism in white adipocytes.

**Figure 1:**
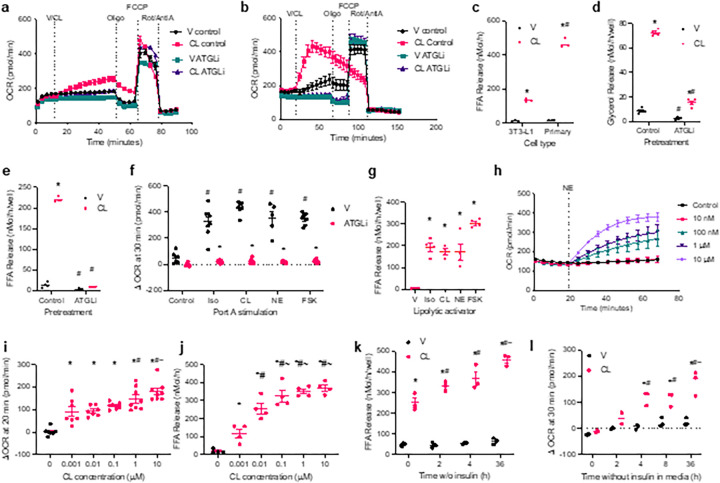
Lipolysis-driven oxidative metabolism in white adipocytes. **a** and **b**, Oxygen consumption rate (OCR) in differentiated 3T3-L1 adipocytes n = 6 (a) and primary adipocytes n= 12 (b) pretreated with 50 mM atglistatin (ATGLi) or vehicle control. Port injection times indicated by vertical lines. CL-316,243 (CL) treatments were 10 mM (a) and 100 nM (b). p-val < 0.05 V control vs. CL control, V control vs V ATGLi, and CL control vs. CL ATGLi during CL and Oligo treatments. **c**, Rate of free fatty acid (FFA) release from 3T3-L1 and primary adipocytes treated with vehicle or 10 mM CL. * p-val < 0.05 V versus CL, ^#^ p-val < 0.05 primary versus 3T3-L1. n = 3. **d**, Rate of glycerol release and **e**, rate of fatty acid release from primary adipocytes pretreated with 50 mM ATGLi or vehicle control, then stimulated with 100 nM CL or vehicle control. * p-val < 0.05 V versus CL, ^#^ p-val < 0.05 control versus ATGLi. n = 4. **f**, Change in OCR in primary adipocytes pretreated with 50 mM ATGLi or vehicle control from baseline to 30 minutes after stimulation with vehicle (control), 1 mM isoproterenol (Iso), 500 nM CL, 10 mM norepinephrine (NE), or 10 mM forskolin (FSK). n = 5–6. **g**, Rate of FFA release from primary adipocytes treated with vehicle, Iso, CL, NE or FSK. n = 4. **h**, OCR in primary adipocytes at baseline and after injection with the indicated concentrations. p-val < 0.05 for all but 10 nM NE versus control. n = 6. **i**, Change in OCR in primary adipocytes baseline to 20 minutes after stimulation with CL at the indicated doses. *p-val < 0.05 versus untreated (0) control, ^#^p-val < 0.05 control versus 1 nM CL, ^~^p-val < 0.05 versus 10 nM and 100 nM CL. N = 7–8. **j**, Rate of FFA release from primary adipocytes after stimulation with CL at the indicated doses. *p-val < 0.05 versus untreated (0) control, ^#^p-val < 0.05 control versus 1 nM CL, ^~^p-val < 0.05 versus 10 nM and 100 nM CL. n = 4. **k**, Rate of FFA release from primary adipocytes stimulation with 100 nM CL or vehicle control after varying times in insulin free media. *p-val < 0.05 V vs. CL, ^#^p-val < 0.05 vs. 0 h, ^~^p-val < 0.05 vs. 2 h. n = 4. and **l**, Change in OCR from baseline to 30 minutes after stimulation with 100 nM CL or vehicle control in primary adipocytes after varying times in insulin free media. *p-val < 0.05 CL vs. V, #p-val < 0.05 vs. 0 h, ^~^p-val < 0.05 vs. 2 h. n = 3.

Stimulation of lipolysis has been previously reported to increase the rate of oxidative metabolism in white adipocytes^9,10,14^. Treatment of 3T3-L1 adipocytes with the β−3 adrenergic receptor agonist CL-316,243 increased oxidative metabolism (Fig. 1a). This increase in oxidative metabolism was dependent on lipolysis, as treatment with the adipose tissue TG lipase (ATGL) inhibitor atglistatin blocked this effect. Moreover, primary adipocyte precursor cells differentiated in vitro exhibited an even more robust increase in oxidative metabolism in response to CL-316,243 (Fig. 1b). While the maximal respiratory capacity of both types of adipocytes is comparable, primary adipocytes are more lipolytically active than 3T3-L1 adipocytes (Fig. 1c). Primary adipocytes reached maximal respiratory capacity 20 minutes after lipolytic stimulation (Fig. 1b). Atglistatin treatment inhibits both stimulated and basal lipolysis in primary adipocytes (Fig. 1d and e). Primary adipocytes also exhibit atglistatin-sensitive oxygen consumption without lipolytic stimulation, attributable to basal rates of lipolysis (Fig. 1b). The increase in lipolysis-driven oxidative metabolism and oxygen consumption was observed with a variety of lipolytic activators, including the non-specific β-adrenergic agonists isoproterenol and norepinephrine as well as the adenylyl cyclase activator forskolin (Fig. 1f and g). The induction of oxygen consumption was dose-dependent, corresponding to the rate of lipolysis (Fig. 1h–j). As expected, insulin attenuated lipolysis and the induction of oxidative metabolism (Fig. 1k and l).

### Intracellular fatty acid levels drive oxidative metabolism

**Figure 2:**
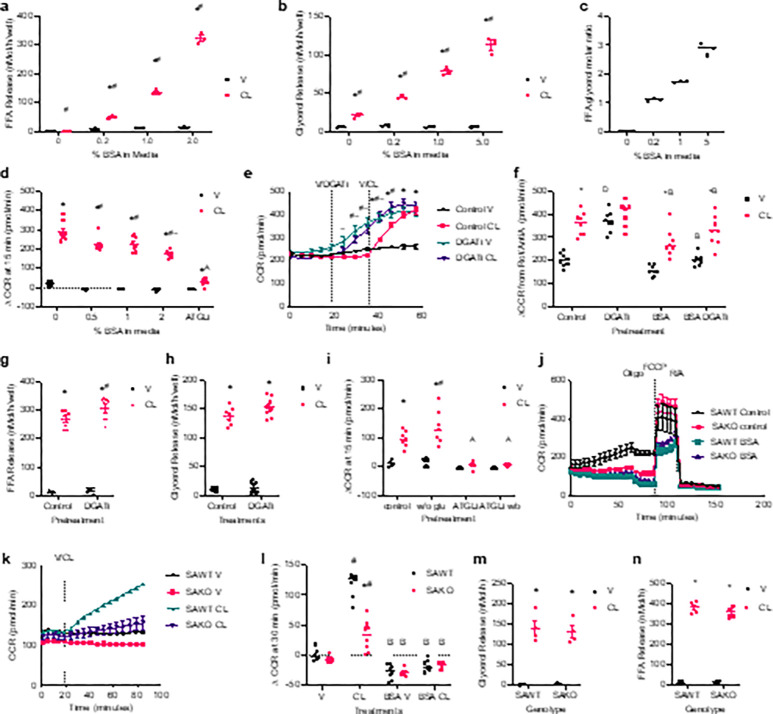
Fatty acid esterification antagonizes oxidative metabolism. **a** and **b**, the rate of FFA (a) and glycerol (b) release in primary adipocytes treated with vehicle or 100 nM CL-316,243 (CL) in media containing varying concentrations of bovine serum albumin (BSA). *p-val < 0.05 CL vs. V, ^#^p-val < 0.05 vs. all other BSA concentrations. **c**, Ratio of fatty acid to glycerol release (from a and b) all BSA concentrations are significantly different from each other, p-val < 0.05. **d**, Change in OCR from baseline to 30 minutes after stimulation with 100 nM CL or vehicle control in primary adipocytes with varying concentrations of BSA in the media or 50 mM ATGLi without BSA. *p-val < 0.05 V vs. CL, ^#^p-val < 0.05 vs. no (0%) BSA, ^~^ p-val < 0.05 vs. 1% BSA media, ^A^p-val < 0.05 ATGLi vs. all BSA concentrations. n = 3. n = 6. **e**, OCR in primary adipocytes; port A: 10 μM PF-04620110 (DGATi) or vehicle control and port B: 50 nM CL vehicle control injected at indicated time points. *p-val < 0.05 V control vs. CL control, ^#^p-val < 0.05 V control vs V DGATi, ~ p-val < 0.05 CL control vs. CL DGATi. n = 8. **f**, Rotenone/antimycin A subtracted OCR after 30 minutes DGATi treatments and 15 minutes 100 nM CL in the presence or absence of 2% BSA. *p-val < 0.05 V vs. CL, ^D^p-val < 0.05 DGATi treatment, ^B^p-val < 0.05 BSA in media. n = 8. **g** and **h**, the rate of FFA (g) and glycerol (h) release in primary adipocytes treated with vehicle or 100 nM CL-316,243 (CL) in after pretreatments with DGATi or vehicle control. *p-val < 0.05 V vs. CL, ^#^p-val < 0.05 control vs. DGATi. n = 7 control and 8 DGATi. **i**, Change in OCR from baseline to 15 minutes after stimulation with 100 nM CL or vehicle control in primary adipocytes pretreated with and without (w/o) glucose (glu) and 50 mM ATGLi or vehicle control. *p-val < 0.05 V vs. CL, ^#^p-val < 0.05 with glucose control, ^A^p-val < 0.05 ATGLi vs. control. n = 7. **j** and **k**, OCR in primary adipocytes isolated from SAKO and litter mate control SAWT mice. **j**, Treatment times 2 mM oligomycin (oligo), 1 mM FCCP, and 1 mM rotenone/antimycin A (R/A). p-val < 0.05 SAWT control vs. SAKO Control, and V SAWT control vs. SAWT BSA, before and after oligo treatment; p-val < 0.05 BSA vs. control, during FCCP treatment. n = 6–8. **k**, Vehicle or 1 nM CL injected at the indicated time. p-val < 0.05 SAKO V vs. SAKO CL, SAWT V control vs. SAKO V, SAKO V vs. SAKO CL, and SAWT V vs. SAWT CL. n = 8. **l**, Change in OCR from baseline to 30 minutes after stimulation with vehicle or 1 nM CL in the presence or absence of 2% BSA in the media. n = 7–8. *p-val < 0.05 SAWT vs. SAKO, ^#^p-val < 0.05 V vs. CL, ^A^p-val < 0.05 +/− BSA. **m** and **n**, the rate of glycerol (m) and FFA (n) release in SAWT and SAKO primary adipocytes treated with vehicle or 100 nM CL. *p-val < 0.05 V vs. CL.

After activation to fatty-acyl CoA, fatty acids can either be esterified via the glycerol lipid synthesis pathway that produces TGs or undergo β-oxidation. Addition of albumin to the media sequesters fatty acids reducing intracellular levels, while in its absence all fatty acids are taken back up by the adipocytes (Fig. 2a). The rate of lipolysis is increased with media BSA (Fig. 2b), due to feedback inhibition of lipolysis by long-chain fatty acids when not buffered by BSA sequestration^13,17–20^. Fatty acid sequestration by BSA in the media modulates the released fatty acid to glycerol ratio (Fig. 2c). Despite increasing lipolytic rates, BSA dose dependently reduces oxidative metabolism during lipolytic stimulation (Fig. 2d), demonstrating that intracellular fatty acid levels drive oxidative metabolism.

#### Fatty acid re-esterification suppresses fatty acid oxidation.

To examine the interplay between re-esterification and oxidative metabolism, we inhibited TG synthesis with the diacylglycerol acyltransferase 1 (DGAT1) inhibitor PF-04620110, effectively blocking TG cycling. Interestingly, inhibition of DGAT1 increased oxygen consumption even in the absence of lipolytic stimulation (Fig. 2e), showing that ATP demand for TG cycling is not the driver of oxidative metabolism. These results suggest that when adipocytes are overburdened with fatty acids that cannot be stored as TGs, fatty acid oxidation is increased, consistent with previous observations^10,21^. Further supporting this notion, sequestration of excess fatty acids by BSA in the media pre-empts the positive impact of DGAT1 inhibition on oxidative metabolism (Fig. 2f). Importantly, DGAT1 inhibition did not affect the rate of basal or stimulated lipolysis, although fatty acid release during stimulated lipolysis was slightly higher (Fig. 2g and h). White adipocytes do not express glycerol kinase but rather derive glycerol-3-phosphate for esterification from glucose metabolism^22,23^. Thus, we also investigated the impact of removing glucose from the media as a means of limiting re-esterification. Lipolysis-driven oxidative metabolism was significantly higher in no glucose media (Fig. 2i), consistent with diversion of fatty acids from re-esterification to oxidative metabolism.

The suppression of re-esterification and diversion of fatty acids into the oxidative pathways is achieved via STAT3 mediated inhibition of GPAT3, the first and rate limiting step in glycerol lipid synthesis^10^. *Stat3* KO adipocytes treated with CL-316,243 showed less lipolysis-driven oxidative metabolism and a significantly lower rate of oxygen consumption compared to wild-type (WT) adipocytes (Fig. 2j and k). Sequestration of fatty acids by BSA eliminated the impact of STAT3 on lipolysis-driven oxidative metabolism (Fig. 2j and l). Importantly, STAT3 did not impact the rate of lipolysis, only the fate of fatty acids within adipocytes (Fig. 2m and n). Together these data demonstrate that when intracellular fatty acid levels are elevated and re-esterification is blocked in white adipocytes, oxidative metabolism increases. This occurs during lipolysis due to flooding of white adipocytes with fatty acids and suppression of glycerol lipid synthesis by STAT3.

#### White adipocyte mitochondria are uncoupled by fatty acids during lipolysis.

**Figure 3:**
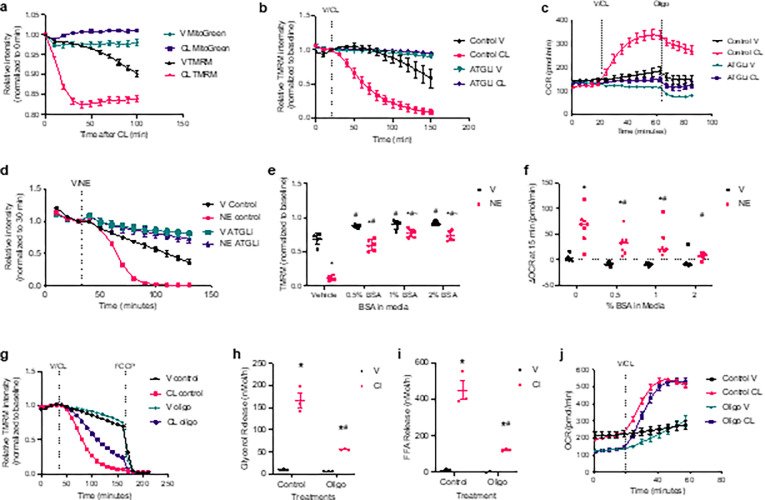
Dissipation of proton motive force during lipolysis. **a**, Baseline normalized TMRM and MitoTracker Green (MitoGreen) staining in primary adipocytes stimulated with 1 mM CL-316,243 (CL) or vehicle control (V). p-val < 0.05 vs. baseline CL TMRM all timepoint, and V TMRM after 60 minutes, no significant change in MitoGreen. n = 6. **b**, Baseline normalized TMRM staining in 50 mM ATGLi and control pretreated primary adipocytes stimulated with 100 nM CL or vehicle control. p-val < 0.05 V control vs. CL control, and CL control vs. CL ATGLi after 40 minutes; p-val < 0.05 V control vs V ATGLi last 30 minutes. n = 24. **c**, OCR in primary adipocytes pretreated with ATGLi or vehicle control, 100 nM CL injected in port A, and 2 mM oligomycin (oligo) injected in port B. p-val < 0.05 V control vs. CL control, V control vs V ATGLi, and CL control vs. CL ATGLi during CL and oligo treatments; and p-val < 0.05 V ATGLi vs. CL ATGLi during oligo treatment and last timepoint of CL. n = 6–8. **d**, Baseline normalized TMRM staining in ATGLi and control pretreated primary adipocytes stimulated with 10 mM norepinephrine (NE) or vehicle control. p-val < 0.05 V control vs. NE control, V control vs V ATGLi, NE control vs. NE ATGLi after NE/V treatment. n = 6. **e**, Baseline normalized TMRM staining in primary adipocytes after 50-minute stimulation with 10 mM NE or vehicle control in varying BSA concentrations. *p-val < 0.05 V vs NE, ^#^p-val < 0.05 vs. control, ^~^p-val < 0.05 vs 0.5% BSA. **f**, Change in OCR from baseline to 15 minutes after stimulation with 10 mM NE or V in varying concentrations of BSA. *p-val < 0.05 V vs NE, ^#^p-val < 0.05 vs. control, **g**, Baseline normalized TMRM staining in primary adipocytes pretreated with 2 mM oligomycin (oligo) or V, 100 nM CL and FCCP treatments at indicated timepoints. Pink: p-val < 0.05 V control vs. CL control, black: p-val < 0.05 V control vs V oligo, purple: p-val < 0.05 CL control vs. CL oligo, and teal: p-val < 0.05 V oligo vs. CL oligo at the time points beneath the line. n = 24. **h** and **i**, the rate of glycerol (h) and FFA (i) release in primary adipocytes treated with vehicle or 100 nM CL after 2 mM oligo pretreatment. *p-val < 0.05 V vs. CL, ^#^p-val < 0.05 oligo vs. control. n = 3. **j**, OCR in primary adipocytes pretreated with 2 mM oligomycin (oligo) or V then stimulated with 100 nM CL or V in port A. p-val < 0.05 V control vs V oligo, and p-val < 0.05 CL control vs. CL oligo at baseline and first two time points after V/CL; and p-val < 0.05 V control vs. CL control V oligo vs. CL oligo after V/CL treatment.

Mitochondrial membrane potential decreased drastically upon lipolytic activation, as determined by staining with the membrane potential-dependent dye tetramethylrhodamine methyl ester perchlorate (TMRM) (Fig. 3a, Extended data 1). The loss of membrane potential requires lipolysis and was blocked by ATGL inhibition (Fig. 3b). Although slower than stimulated cells, vehicle control cells also lost membrane potential over time in an ATGL-dependent manner. While membrane potential was dissipated, oxygen consumption rates increased (Fig. 3b and c). This rapid dissipation of the proton motive force is suggestive of uncoupled respiration. Consistent with uncoupling, lipolysis-driven oxidative metabolism was insensitive to the ATP synthase inhibitor oligomycin (Fig. 3c). Norepinephrine also induced a lipolysis-dependent loss of mitochondrial membrane potential (Fig. 2d). Sequestration of fatty acids by BSA in the media attenuated the loss of membrane potential and reduced the rate of oxygen consumption (Fig. 3e and f). These data suggest that the elevation in intracellular fatty acids mediates mitochondrial depolarization in lipolytic adipocytes.

During coupled respiration, ATP synthase utilizes the proton motive force to drive ATP production. To investigate the role of ATP synthase in the loss of proton motive force, we pretreated cells with oligomycin to inhibit ATP synthesis. Inhibition of ATP synthase slowed the loss of membrane potential (Fig. 3g). Pretreatment with oligomycin also reduced the rate of lipolysis (Fig. 3h and i), as previously observed^24^. Thus, it is not clear if ATP synthesis contributes to the loss of membrane potential or if the reduction in fatty acid load slowed the loss of membrane potential. Nevertheless, significant membrane depolarization was observed in the presence of oligomycin, strongly indicating a proton leak and uncoupled respiration (Fig. 3g). We also investigated the effect of oligomycin pretreatment on lipolysis-driven oxidative metabolism. As expected, oligomycin reduced baseline oxygen consumption; however, oxygen consumption rapidly increased upon CL-316,243 stimulation in the oligomycin-treated adipocytes, catching up with the CL-316,243-treated control adipocytes (Fig. 3j). This suggests that ATP utilization is not the driving force behind the increase in oxidative metabolism, but rather a proton-leak-dependent reduction in membrane potential that facilitates electron transport, increased oxygen consumption and upstream substrate utilization.

#### What is the mechanism of uncoupling in white adipocytes?

In brown adipocytes, uncoupled respiration occurs due to activation of UCP1 by fatty acids^25–28^. Although Ucp1 expression in primary white adipocytes is quite low, we used high concentrations of GDP to inhibit any possible UCP1 activity. The basal oxygen consumption rate was lower in the presence of GDP (Extended Data 2a). Upon stimulation, the oxygen consumption rate increased rapidly, reaching CL-316,243 control levels. The effect of GDP was similar to oligomycin, consistent with inhibition of ATP synthase^29^. To investigate the specific effect of GDP on uncoupled respiration, we examined its effect in combination with oligomycin. GDP attenuated the loss of membrane potential, but the effect was less than that of oligomycin, and GDP had no additional impact on membrane potential in the context of oligomycin treatment (Extended Data 2b). These findings are consistent with previous reports that fatty acid mediated uncoupling in lipolytic adipocytes is independent of UCP1^14^.

**Figure 4:**
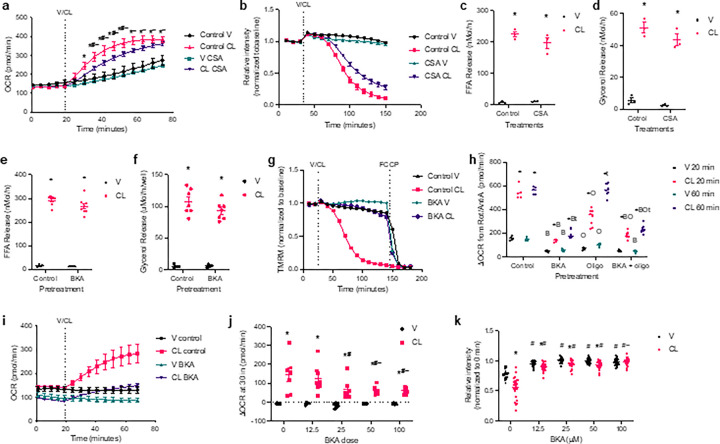
Fatty acids promote uncoupling via AAC in white adipocytes. **a**, OCR in primary adipocytes pretreated with 50 mg/mL Cyclosporin A (CSA) or V for 24 hours, then stimulated with 100 nM CL or V in port A. Pink: p-val < 0.05 V control vs. CL control, purple: p-val < 0.05 CL control vs. CL CSA, and teal: p-val < 0.05 V CSA vs. CL CSA at the time points beneath the line. n = 5–6. **b**, Baseline normalized TMRM staining in primary adipocytes pretreated with 50 mg/mL CSA or V for 24 hours and stimulated with 100 nM CL or V. *p-val < 0.05 V control vs. CL control, ^#^p-val < 0.05 CL control vs. CL CSA, and ^~^p-val < 0.5, V CSA vs. CL CSA at the time points beneath the line. n = 24. **c** and **d**, the rate of FFA (c) and glycerol (d) release in primary adipocytes pretreated with vehicle or 50 mg/mL CSA or V for 24 hours, then 100 nM CL. *p-val < 0.05 V vs. CL. n = 3. **e** and **f**, the rate of FFA (e) and glycerol (f) release in primary adipocytes pretreated with vehicle or 100 mM bongkrekic acid (BKA) or V then 100 nM CL. *p-val < 0.05 V vs. CL. n = 4/V and 7/CL. **g**, Baseline normalized TMRM staining in primary adipocytes pretreated with 100 mM BKA or V, then 100 nM CL and FCCP treatments at indicated timepoints. p-val < 0.05 V control vs. CL control, V control vs V BKA, CL control vs. CL BKA, and V BKA vs. CL BKA during V/CL treatment. n = 24. **h**, OCR in in primary adipocytes pretreated +/− 100 mM BKA and 2 mM oligo 20 and 60 minutes after stimulation with vehicle or 50 nM CL, ^#^p-val < 0.05 V vs. CL, ^B^p-val < 0.05 BKA vs. control. ^O^p-val < 0.05 Oligo vs. control. ^t^p-val < 0.05 20 vs. 60 min. n = 5–8. **i**, OCR in in primary adipocytes pretreated with 100 mM BKA then stimulated with V or 50 nM CL. p-val < 0.05 V control vs. CL control, CL control vs. CL BKA, and V BKA vs. CL BKA after V/CL treatment. n = 8. **j**, Change in OCR from baseline to 30 min after stimulation with 50 nM CL or V in primary adipocytes pretreated with BKA then stimulated with V or 50 nM CL. *p-val < 0.05 V vs. CL, ^#^p-val < 0.05 vs no BKA control, ^~^p-val < 0.05 vs. 12.5 mM BKA. n = 4–8. **k**, Baseline normalized TMRM staining in primary adipocytes pretreated with BKA then stimulated with V or 50 nM CL. *p-val < 0.05 V vs. CL, ^#^p-val < 0.05 vs no BKA control, ^~^p-val < 0.05 vs. 12.5 mM BKA. n = 23–24.

The permeability transition pore (PTP) is a large multiprotein complex in the mitochondrial inner membrane that is defined by the presence of cyclophilin D^30–32^. PTP opening can result in cytochrome C release, mitochondrial swelling, and cell death. However, transient opening of the PTP also occurs physiologically^33,34^, and is a potential mechanism by which uncoupling could occur in lipolytic white adipocytes^9^. Inhibition of cyclophilin D with cyclosporin A (CSA) ^35^ attenuates the loss of membrane potential and the increase in lipolysis-driven oxidative metabolism but does not impact the rate of lipolysis (Fig. 4a–d). While significant, the effect of CSA was small, and membrane potential dropped while oxygen consumption increased. The PTP is activated by calcium signalling, thus we investigated the impact of ruthenium red, which blocks mitochondrial calcium flux^36^. Ruthenium red had only a small impact on membrane potential loss and did not impact oxygen consumption (Extended Data 2c and d).

A common component of the PTP is AAC^33^. Like UCP1, AAC nucleotide binding can be outcompeted by fatty acids leading to a proton leak^15,16^. Given that lipolysis-driven uncoupling appears to be mediated by fatty acids, we investigated the impact of inhibiting AAC with bongkrekic acid (BKA) ^37^. Treatment with BKA had no significant impact on the rate of lipolysis, but blocked membrane depolarization (Fig. 4e–g). Since AAC activity is required to provide ADP for ATP synthesis, BKA inhibited basal oxygen consumption to the same extent as oligomycin, and the two inhibitors together had no additive effect on basal respiration (Fig. 4h). However, upon lipolytic stimulation, BKA treatment blocked the increase in oxygen consumption (Fig. 4h and i). Even in the presence of oligomycin, BKA significantly reduced lipolysis-stimulated oxidative metabolism (Fig. 4h). The effect of BKA to attenuate the induction of oxidative metabolism was dose-dependent (Fig. 4j). BKA efficiently blocked the loss of mitochondrial membrane potential at all concentrations tested (Fig. 4k). These data suggest that fatty acid binding to AAC is responsible for the proton leak that leads to the loss of mitochondrial membrane potential and drives oxidative metabolism. Colour

#### Uncoupled respiration has greater thermogenic potential than TG cycling.

All metabolism generates heat in order to be thermodynamically favourable. Full β-oxidation of fatty acids is about 40% efficient (producing ATP), while the remaining chemical energy is converted to heat. Thermogenic reactions produce heat via futile cycles that drive upstream metabolic pathways. In the white adipose tissue futile TG cycling consumes 7 ATP per cycle. We propose that uncoupled oxidation of fatty acids in lipolytic adipocytes is an additional thermogenic pathway. Six of the seven ATP consumed by TG cycling are used to convert fatty acids into fatty acyl-CoAs, which is also required for β-oxidation. When coupled, full oxidation of palmitoyl-CoA generates 131 ATP. Uncoupling via AAC would be predicted to waste up to 399 ATP (3 × 131 + 6) per TG. Thus, thermogenesis in white adipocytes during lipolysis could be driven more robustly by uncoupled fatty acid oxidation than TG cycling.

**Figure 5:**
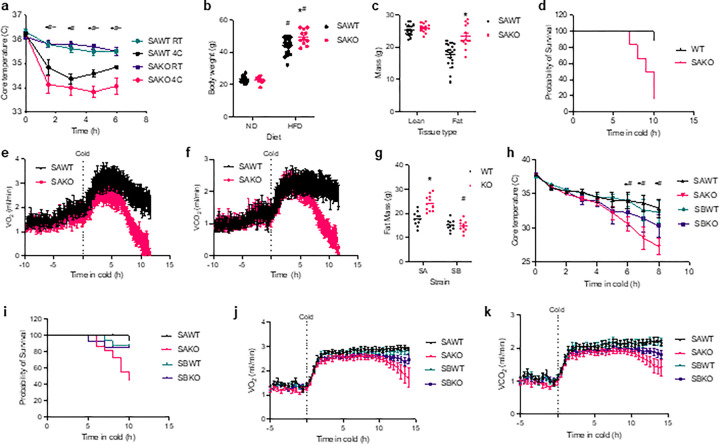
Obese thermoneutral-adapted SAKO mice exhibit a defect in cold tolerance. **a**, Core body temperature during acute cold exposure (4C) or room temperature controls (RT) in obese male SAKO and SAWT. *p-val < 0.05 SAWT 4C vs. SAKO 4C, ^#^p-val < 0.05 SAWT RT vs SAWT 4C, and ^~^p-val < 0.05 V SAKO RT vs. SAKO 4C at the time points beneath the line. n = 8/RT and 7/4C. **b**, Body weight in male normal diet (ND) and high fat diet (HFD) fed SAWT and SAKO mice. *p-val < 0.05 SAWT vs. SAKO, ^#^p-val < 0.05 NV vs. HFD. n = 8–23 mice. **c**, Lean and fat mass in male obese SAWT and SAKO mice. *p-val < 0.05 SAWT vs. SAKO. n = 21 SAWT and 13 SAKO mice. **d**, Survival curve for obese thermoneutral-adapted male SAWT and SAKO mice during cold exposure. n = 9 SAWT and 6 SAKO mice. **e** and **f**, Oxygen consumption (VO_2_) (e) and carbon dioxide production (VCO_2_) (f) of obese thermoneutral-adapted male SAWT and SAKO mice at 30 °C then transitioned to 5 °C (cold) at time zero. p-val < 0.05 SAWT vs. SAKO. n = 15 SAWT and 9 SAKO. **g**, Fat mass in obese thermoneutral-adapted male SAWT/KO and SBWT/KO mice. *p-val < 0.05 WT vs. KO. ^#^p-val < 0.05 SA vs. SB. **h**, Core body temperature during acute cold exposure in male obese thermoneutral-adapted SAWT/KO and SBWT/KO mice. *p-val < 0.05 SAWT vs. SAKO, and ^#^p-val < 0.05 SAKO RT vs SBKO 4C at the time points beneath the line. n = 6. **i**, Survival curve for obese thermoneutral-adapted male SAWT/KO and SBWT/KKO mice during cold exposure. n = 21 SAWT, 20 SAKO, 16 SBWT and 14 SBKO mice. **j** and **k**, VO_2_ (j) and VCO_2_ (k) of obese thermoneutral-adapted male SAWT/KO and SBWT/KO mice at 30 °C then transitioned into cold at time zero. p-val < 0.05 SAWT vs. SAKO last six timepoint, SAKO RT vs SBKO 4 Clast 4 timepoints, and SBWT vs. SBKO last two time points of k. n = 9–13.

To test this concept, we investigated cold tolerance in adipocyte-specific *Stat3* knockout (SAKO) mice, which, due to the loss of STAT3 mediated inhibition of GPAT3, exhibit more TG cycling and less β-oxidation during lipolysis in white adipocytes^10^. Normal diet SAKO mice were found to have normal cold tolerance and energy expenditure at both 22 °C and 5 °C (Extended Data 3). However, when placed on a high-fat diet, SAKO mice were cold-sensitive as compared to their littermate controls (Fig. 5a). To further focus on the specific metabolic contributions of white adipocytes, we adapted the mice to thermoneutrality, inactivating their brown adipose tissue. The obese thermoneutral-adapted SAKO mice had increased body weight due to increased adiposity (Fig. 5b and c), as was previously observed in diet induced obese mice housed at room temperature^10^. The cold sensitivity of the SAKO mice was enhanced by thermoneutral adaptation, resulting in a survival defect in the SAKO mice (p-value = 0.003) (Fig. 5d). Initially, oxygen consumption increased in both genotypes, likely due to shivering. However, over time the SAKO mice failed to maintain this increased oxidative rate and the oxygen consumption dropped lower than in SAWT controls (Fig. 5e). The same pattern of carbon dioxide production was observed (Fig. 5f). No differences in respiratory exchange ratio, activity or food intake were observed (Extended Data 4). Taken together these data indicate that obese thermoneutral-adapted SAKO mice exhibit increased sensitivity to acute cold exposure, failing to mount the respiratory response required for survival in these conditions.

#### STAT3 is required for thermogenesis in UCP1-null adipocytes.

*Stat3* is knocked out in all adipocytes by *Adipoq*-CRE. To investigate the contribution of classic thermogenic adipocytes, we utilized a *Ucp1* promoter-driven CRE to knock *Stat3* out in brown adipocytes (SBKO mice). Like SAKO mice, normal diet room temperature housed SBKO mice exhibited no defect in cold-induced energy expenditure (Extended Data 5a and b). However, while obese thermoneutral-adapted SAKO mice exhibit cold sensitivity, SBKO mice have normal cold tolerance and energy expenditure (Extended Data 5c-f). Obese thermoneutral-adapted SBKO mice did not exhibit the increase in adiposity observed in SAKO mice (Fig. 5g). To compare the SAKO and SBKO phenotypes we generated a large cohort with both genotypes and their corresponding controls. In an acute cold tolerance test, the SAWT and SBWT control mice defended their core temperature similarly, while the core temperature of the SAKO mice dropped significantly lower than the SAWT and SBKO animals (Fig. 5h). Furthermore, SBKO mice did not exhibit a survival defect as compared to the SBWT controls (p-value = 0.86), unlike the SAKO versus SAWT mice (p-value = 0.0004) (Fig. 5i). During acute cold exposure, oxygen consumption in SBKO mice was not significantly different from their SBWT littermate controls, while oxygen consumption in SAKO mice was significantly lower than both SAWT and SBKO mice (Fig. 5j). Carbon dioxide production in the SBKO mice dropped lower than the SBWT controls; however, the rate in the SAKO mice dropped earlier and was significantly lower than in the SBKO mice (Fig. 5k). There were no differences in respiratory exchange ratio or physical activity between the genotypes (Extended Data 6). Additionally, there were no significant differences in thermogenic gene expression in inguinal white or epididymal white adipose tissue (Extended Data 7). Overall, these data indicate that *Stat3* knockout in white adipocytes causes increased sensitivity to cold in obese thermoneutral-adapted mice, which is associated with a defect in energy expenditure in the cold. Knockout of *Stat3* in brown adipocytes had no impact on thermogenesis regardless of body composition or housing temperature.

#### Lipolysis-driven thermogenesis is independent of physical activity.

**Figure 6:**
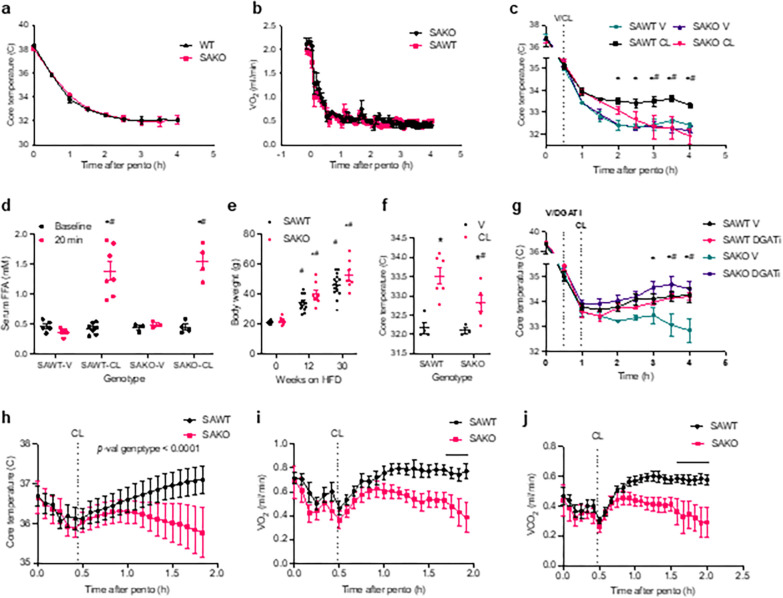
Lipolysis-driven thermogenesis is independent of physical activity. **a** and **b**, Core body temperature (a) and Oxygen consumption (VO_2_) (b) in obese thermoneutral-adapted male SAWT and SAKO injected with pentobarbital (pento) at time zero to block muscle activity. **c**, Core body temperature in obese thermoneutral-adapted male mice injected with pentobarbital at time zero and 3 mg/kg DGATi or vehicle control at 30 minutes, all mice received 1 mg/kg CL at 60 minutes. ^#^p-val < 0.05 SAWT V vs. SAKO V and *SAKO V vs SAKO DGATi. **d**, Core body temperature in obese thermoneutral-adapted male SAWT and SAKO injected with pentobarbital at time zero, and 1 mg/kg CL or vehicle control at 30 minutes. Pink: SAWT CL vs SAKO CL, black: p-val < 0.05 SAWT V vs. SAWT CL at the time points beneath the line. **e**, Serum free fatty acid (FFA) levels at baseline and 20 minutes after V and CL injection in obese thermoneutral-adapted male SAWT and SAKO mice. *p-val < 0.05 baseline vs. 20 min. ^#^p-val < 0.05 V vs. CL. n = 3 to 7 mice. **f**, Body weight of female SAWT and SAKO mice on HFD. *p-val < 0.05 SAWT vs. SAKO. ^#^p-val < 0.05 vs. previous time point. n = 14 SAWT and 8 SAKO. **g**, Core body temperature in obese thermoneutral-adapted female SAWT and SAKO injected with pentobarbital, 2 hours after injection of 1 mg/kg CL or vehicle control. n = 3–5. **h-j**, Core body temperature (h), VO_2_ (i) and VCO_2_ (j) of obese thermoneutral-adapted male SAWT and SAKO with implanted temperature probes in metabolic cages and injected with 1 mg/kg CL. p-val < 0.05 SAWT vs. SAKO last 3(i) and 6(j) time points. n = 6 SAWT and 5 SAKO.

Physical activity generates heat, and shivering contributes to energy expenditure during acute cold exposure. To isolate the effect of lipolysis-driven uncoupled respiration on thermogenesis, we blocked muscle activity in addition to thermoneutral housing and a high fat diet. Even at 30 °C, when mice are immobilized with pentobarbital their core body temperature declines dramatically before it levels off at 32–33 °C (Fig. 6a). Core body temperature and energy expenditure dropped equally in SAWT and SAKO mice upon immobilization (Fig. 6a and b). Injection of CL-316,243 after immobilization protects core body temperature in obese thermoneutral-adapted mice (Fig. 6c). While core body temperature was significantly higher in SAWT CL-316,243-injected mice as compared to vehicle controls, CL-316,243 did not have a significant impact on temperature in the SAKO mice, whose temperature was significantly lower than the SAWT CL-316,243-treated mice (Fig. 6c). Importantly, the lipolytic response to CL-316,243 in the SAKO mice was not defective; serum fatty acid levels were increased equally in the SAWT and SAKO mice treated with CL-316,243 (Fig. 6d). Female mice were placed on a high-fat diet for 30 weeks to achieve weight gain comparable to males (Fig. 6e). A defect in CL-316,243-induced thermogenesis was also observed in female SAKO mice relative to SAWT controls (Fig. 6f). The thermogenic defect in the SAKO mice was corrected by pretreatment with the DGAT1 inhibitor PF-04620110 (Fig. 6g), consistent with STAT3-mediated repression of esterification as the mechanism of induction of oxidative metabolism and thermogenesis.

A thermal probe was implanted into a cohort of SAWT and SAKO mice to obtain simultaneous core temperature and energy expenditure readings. After pentobarbital injection, core temperature and oxidative metabolism were reduced similarly in SAWT and SAKO mice (Fig. 6h–j). However, the two genotypes diverged upon CL-316,243 injection, which resulted in significantly higher core body temperature, oxygen consumption, and carbon dioxide production in the SAWT compared to the SAKO mice (Fig. 6h–j). These data suggest that the thermogenic effect of CL-316,243 in obese thermoneutral-adapted mice is dependent on adipocyte STAT3 but independent of muscle activity, consistent with a thermogenic effect of lipolysis-driven oxidative metabolism in white adipocytes.

## Discussion

Our data suggest that during lipolysis, fatty acids activate the uncoupling function of AAC in white adipocytes, driving oxidative metabolism and contributing to whole-body energy expenditure. This represents a crucial thermogenic pathway in obese thermoneutral-adapted mice. These findings challenge the dogma that uncoupled respiration in adipocytes is mediated by UCP1, and thus limited to UCP1-expressing adipocytes. While ATP-dependent futile cycles have been demonstrated to contribute to thermogenesis, a physiological UCP1-independent uncoupling mechanism has not been elucidated in white adipocytes. Our data suggest that fatty acid-activated uncoupling is a universal property mediated by AAC in white adipocytes.

Nucleotide exchange by the ATP/ADP carrier is required to supply ADP for coupled respiration; thus, AAC knockout blocks coupled respiration as well. Mice express two somatic AAC isoforms. AAC1 (*Slc25a4*) expression is highest in the heart and skeletal muscle, and its knockout results in heart failure^38,39^. AAC2 (*Slc24a5*) is broadly expressed and whole-body knockout is embryonically lethal^39,40^. Humans also express AAC3 (*SLC25A6*) and exhibit different tissue distributions of ACCs^39,41^. All somatic isoforms are expressed in adipocytes^9,42^. Adipocyte-specific knockout of *Slc25a5* reduces adipocyte oxygen consumption leading to increased adipocyte size and adiposity^43^. However, the metabolic and thermogenic response to catecholamine stimulation has not been studied in these animals. The *Slc25a5* floxed strain is currently cryopreserved at IKMC and could be useful in future studies upon recovery. However, due to high sequence homology, it may be necessary to knockout all AAC isoforms to block uncoupling.

While the lack of an in vivo AAC knockout model is a limitation of this study, we utilized adipocyte-specific *Stat3* KO mice for in vivo validation, as they exhibit a specific defect in fatty acid handling in lipolytic adipocytes without secondary effects on the rate of lipolysis or coupled respiration^10^. Using this model, we were able to demonstrate that fatty acid induced uncoupled oxidative metabolism promotes thermogenesis to a greater extent than TG cycling in white adipocytes. Furthermore, we demonstrated that the thermogenic defect in the SAKO mice can be reversed by inhibition of TG synthesis, consistent with suppression of re-esterification being the mechanism by which STAT3 promotes oxidative metabolism.

Energy expenditure via uncoupled respiration in adipocytes has been of great interest in the treatment of obesity^44–46^. However, the lack of brown adipose tissue in individuals with obesity has been a hurdle to realizing this treatment strategy^47^. The identification of an energy-expending pathway in white adipocytes, opens a new avenue for obesity treatment. Given that obesity is characterized by an overabundance of white adipose tissue, even a small increase in white adipocyte energy expenditure could have a substantial impact on whole-body energy balance. This treatment strategy is complementary to current GLP-1-based therapies, which reduce food intake^48^. A potential pitfall of increasing adipocyte energy expenditure is the possible induction of hypoxia in the adipose tissue, which could negatively impact adipose function^42,43^.

Adipocyte fatty acid handling is of fundamental importance to adipocyte biology. Lipolysis floods adipocytes with fatty acids, significantly changing adipocyte metabolism and whole-body energy balance. We have previously reported that fatty acids stimulate serine 727 phosphorylation of STAT3 promoting its interaction with and repression of GPAT3. Our results suggest that intracellular fatty acids also promote mitochondrial uncoupling through AAC. As a result, lipolysis drives uncoupled respiration in white adipocytes, impacting whole-body energy expenditure and thermogenesis. However, important questions remain: How is fatty acid retention versus release into the circulation regulated in adipocytes? Which fatty acid-binding proteins, transporters and CoA synthetases are involved? How do the proteins involved and their localization impact the activation of AAC by free fatty acids and the metabolic fate of fatty-acyl CoAs? We hope the findings reported here will lay the groundwork for future studies investigating fatty acid handling in adipocytes and its impact on energy balance and metabolic health.

## Methods

### Reagents

The following reagents were used in this study: Amphotericin B (Sigma, A2411)

Antimycin A (Sigma A8674), Atglistatin (ATGLi, Sigma SML1075), Bongkrekic acid (BKA, Enzo BML-CM113), Carbonyl cyanide 4-(trifluoromethoxy)phenylhydrazone (FCCP, Sigma C2920), CL-316,243 (CL, Sigma C5979), Collagenase (Sigma – C6885), Cyclosporin A (CSA, Enzo BML-A195), Dexamethasone (Sigma D4902), DMEM/F-12 50/50 (Corning 15–090), Fetal bovine serum (FBS, Corning 35–010), Extracellular matrix gel from Engelbreth-Holm-Swarm murine sarcoma (Sigma E1270), Fibronectin (Sigma F1141) Forskolin (FSK, Sigma F3917), Guanosine diphosphate (GDP, Sigma G7127), Insulin (Sigma I6634), 3-Isobutyl-1-methylxanthine (IBMX, Sigma I5879), Isoproterenol (Sigma PHR2722), MitoTracker green (Fisher M7514), Norepinephrine (NE, Sigma A7257), Oligomycin A (Sigma 75351), Pen Strep Glutamine (PSG, Gibco 10378–016), Pentobarbital (Covetrus 081799), PF-04620110 (DGATi, Sigma PZ0207), Rosiglitazone (Sigma 557366), Rotenone (Sigma R8875), Tetramethylrhodamine, methyl ester (TMRM, Fisher I34361).

### Animals

Animals homozygous for the *Stat3* floxed allele (Stock No: 016923) were bred to *Adipoq*-promoter driven *Cre* mice (Stock No: 028020) to generate mice homozygous for the *Stat3* floxed allele both with and without the *Adipoq-Cre*. Animals with *Adipoq-Cre* expression lose *Stat3* in mature adipocytes and are referred to in the manuscript as SAKO animals, while floxed littermate controls without *Adipoq-Cre* are referred to as SAWT. Animal homozygous for the *Stat3* floxed allele were also bred to *Ucp1*-promoter driven *Cre* mice (Stock No: 024670) to generate mice homozygous for the *Stat3* floxed allele both with and without the *Ucp1-Cre*. Animals with *Ucp1-Cre* expression model where the *Stat3* gene has been specifically lost in brown adipocytes and are referred to in the manuscript as SBKO animals, while floxed littermate controls without *Ucp1-Cre* are referred to as SBWT.

All strains of mice were on the C57BL/6J background (Stock No: 000664). Animals for experiments were bred in-house. Animals in each cohort were produced from multiple breeding pairs to minimize the birthdate range. Extra attention was paid to housing arrangements to ensure that each cage accommodated multiple treatment groups, to minimize potential confounding by the cage effect. During animal studies, ear tag numbers were used to identify animals. Within an experiment, the genotype and/or treatment groups were both littermates and cage mates. Researchers performing tests and collecting data were blinded during experiments. Sample sizes were determined using a power analysis with the expected effect size but were sometimes limited by availability. Sex as a biological variable was considered; female and male cohorts were analysed separately.

Mice were housed in a specific pathogen-free facility with a 12-hour light-dark cycle and were given free access to food and water. Mice were fed normal diet (5053, Labdiet Picolab) or high-fat diet (HFD) (D12451, Research Diets Inc.) with 45% of calories from fat. Starting dates on the High Fat diet were determined according to gender. Female groups were started on the high-fat diet at 10 to 14 weeks of age, and males at 6 to 10 weeks of age. Thermoneutral adapted mice were housed at 30 °C for a minimum of 10 days. All animal use was approved by the Institutional Animal Care and Use Committee (IACUC) at Weill Cornell Medicine.

#### Body composition (Eco-MRI):

Body composition was determined at the Weill Cornell Medicine Metabolic Phenotyping Center using an EchoMRI^™^ (3 in 1).

#### Indirect calorimetry (metabolic cage experiments):

Mice were housed in Sable Systems International Promethium metabolic cages to measure oxygen consumption (VO ), carbon dioxide production (VCO ) via indirect calorimetry, and spontaneous motor activity at the Weill Cornell Metabolic Phenotyping Center. Cages were situated in environmentally controlled chambers (DB034-LT) for precise temperature control.

#### Cold exposure:

Acute cold exposure (5°C) was performed in a climate-controlled room or environmental chamber. Mice were single housed in standard housing cages or metabolic cages. Rectal temperature measurements were taken using a mouse rectal probe and thermometer (PhysiTemp, BAT-12).

#### Thermogenesis in immobilized mice:

Mice were anesthetized with an intraperitoneal injection of 70 mg/kg pentobarbital. After 30 minutes, the mice were intraperitoneally injected with up to 1 mg/kg CL-316,243 or PBS vehicle, or 3 mg/kg DGATi or vehicle control were intraperitoneally injected at 30 minutes, and 1 mg/kg CL-316,243 at 60 minutes. The depth of anaesthesia was assessed every 15 to 30 minutes by applying noxious stimuli (e.g., the pedal withdrawal reflex in the hind limbs). If any response was observed, 20 to 30 mg/kg pentobarbital was administered.

#### Temperature Probe implantation:

A G2 E-mitter temperature probe (Starr Life Sci. Corp) was implanted in the peritoneal cavity of 6-week-old mice. Mice were anesthetized with Ketamine/Xylazine and sterile lubricant applied to both eyes. The abdomen was shaved, and the surgical site sterilized. A 2 cm midline ventral incision was made 1 cm below the diaphragm. The skin was then retracted, and the abdominal cavity was opened by making a 1 cm incision along the posterior left lower quadrant. The temperature probe was positioned in the abdominal cavity ventral to the digestive organs. Local anaesthetic was applied, and the abdominal cavity was closed using monofilament absorbable sutures and wound clips. Post surgical pain was managed with buprenorphine and meloxicam. Two weeks after the operation, mice were placed on HFD then thermoneutral adapted for the experiment.

#### In vivo lipolysis:

Blood was collected by submandibular bleed, coagulated and centrifuged at 10,000 × g for 10 minutes at 4 °C to separate the serum. For free fatty acid (FFA) measurement, 2 μL of serum was used with the NEFA Linearity Set (Wako’s NEFA HR 2, 999–34691- 991–34891- 995–34791- 993–35191). The assay was performed using 75 μL of Reagent A and 150 μL of Reagent B. Absorbance was measured at 550 nm with a reference wavelength of 660 nm, following the manufacturer’s protocol.

#### Real-time PCR analysis of gene expression:

Adipose tissue was homogenized in Trizol (Invitrogen) and mixed with 20% chloroform. RNA extractions from inguinal WAT, epididymal WAT and BAT were performed using the PureLink^™^ RNA Mini Kit (Thermo Fisher Scientific, 12183020). We used SuperScript IV VILO Master Mix Synthesis System for reverse transcription–PCR (Thermo Fisher Scientific, 11766050) with a 3:1 mixture of random hexamers to oligo dT primers for reverse transcription. Real-time PCR amplification was performed on samples in triplicate with Power SYBR Green PCR Master Mix (Applied Biosystems, A46109) using the Applied Biosystems QuantStudio5 real-time PCR System and quantified using an internal standard curve with *Arbp* as the control gene. The sequences of all primers used in this study are listed in Extended data table 1.

### Cell Culture

#### Primary adipocytes:

Primary preadipocytes were isolated from inguinal fat pads as follows^49^: Following fine mincing, the tissue was digested with 1 mg/ml collagenase and 2% BSA in a 37 °C water bath with shaking for 20–35 min. The digestion was stopped by adding 15% FBS media 1:1 to the serum-free collagenase media, then the slurry was passed through a 100 μm filter and spun at 500 g for 5 min. The pellet was washed and resuspended in culture media (DMEM/F-12 with 15% FBS and PSG) and plated with 2.5 mg/L amphotericin B and placed in a 10% CO_2_ incubator. Nonadherent cells were washed away three days later. When the cells reached ~80 % confluence, they were passaged from their original 10 cm culture plate to a 15 cm plate and incubated for an additional 2–5 days. Cells from the second passage were plated to confluence for experiments on extracellular matrix and fibronectin coated plates. Differentiation was initiated with 500 μM 3-Isobutyl-1-methylxanthine, 5 μM dexamethasone, 1 μg/ml insulin and 1 μM rosiglitazone for 3 days, followed by insulin alone for at least 3 days. Cells were used for experiment 7–10 days after the initiation of differentiation. Twenty-four hours prior to the start of an assay, insulin was removed from the culture media. Only cultures in which >90% of cells displayed adipocyte morphology were used.

#### 3T3-L1 Adipocytes:

3T3-L1 fibroblasts (American Type Culture Collection) were differentiated as previously described^50^.

#### Seahorse respiration assays:

Extracellular oxygen consumption rates were measured with a Seahorse XFe96 analyser. Primary preadipocytes were differentiated in 96 well Seahorse XFe96 culture plates. After differentiation, cells were switched to Seahorse XF base DMEM (Agilent 102353) supplemented with 2 mM glutamine, 1 mM pyruvate, and 8 mM glucose. Pre-treatments were added to the base medium. During the assays, drug treatments were injected sequentially using the ports. Unless otherwise indicated, Port A contained 100 nM CL-316,2 or vehicle control, Port B: 2 mM oligomycin, Port C: 1 mM FCCP, and Port D: 1 mM rotenone and 1 mM antimycin A.

#### Mitochondrial membrane potential:

Tetramethylrhodamine, Methyl Ester, Perchlorate (TMRM) staining was performed and imaged with an imageXpress MICRO Confocal Automated High-Content Analysis System to visualize live mitochondria membrane potential. Cells were stained with 200 nM TMRM thirty minutes, then washed three times with PBS. Imaging was performed in live cell imaging solution (Invitrogen A59688DJ). After baseline images were obtained, cells were treated with either vehicle control or CL-316,243. Finally, cells were treated with 1 mM FCCP as a negative control form membrane potential.

#### Lipolysis assay:

Lipolysis in primary adipocytes was performed as preciously decribed^51^.

### Statistical analysis:

Two-way or one-way analysis of variance (ANOVA) was performed to evaluate statistical significance, followed by the Holm-Sidak post-hoc analysis to determine specific between-group and time-dependent differences. In each case, significance was set at α = 0.05. Statistical analyses were performed in GraphPad Prism version 10.

## Data Availability

Data supporting the findings of this study are included within the manuscript and extended data. The corresponding author will provide data upon request.
